# Immune response treated with bone marrow mesenchymal stromal cells after stroke

**DOI:** 10.3389/fneur.2022.991379

**Published:** 2022-09-20

**Authors:** Zili Wang, Xudong Wang, Yidong Liao, Guangtang Chen, Kaya Xu

**Affiliations:** ^1^Department of Neurosurgery, The Affiliated Hospital of Guizhou Medical University, Guiyang, China; ^2^School of Clinical Medicine, Guizhou Medical University, Guiyang, China

**Keywords:** stroke, bone marrow mesenchymal stromal cell, stem cell therapy, inflammation, neuroprotection

## Abstract

Stroke is a leading cause of death and long-term disability worldwide. Tissue plasminogen activator (tPA) is an effective treatment for ischemic stroke. However, only a small part of patients could benefit from it. Therefore, finding a new treatment is necessary. Bone marrow mesenchymal stromal cells (BMSCs) provide a novel strategy for stroke patients. Now, many patients take stem cells to treat stroke. However, the researches of the precise inflammatory mechanism of cell replacement treatment are still rare. In this review, we summarize the immune response of BMSCs treated to stroke and may provide a new perspective for stem cell therapy.

## Introduction

**Figure 1:**
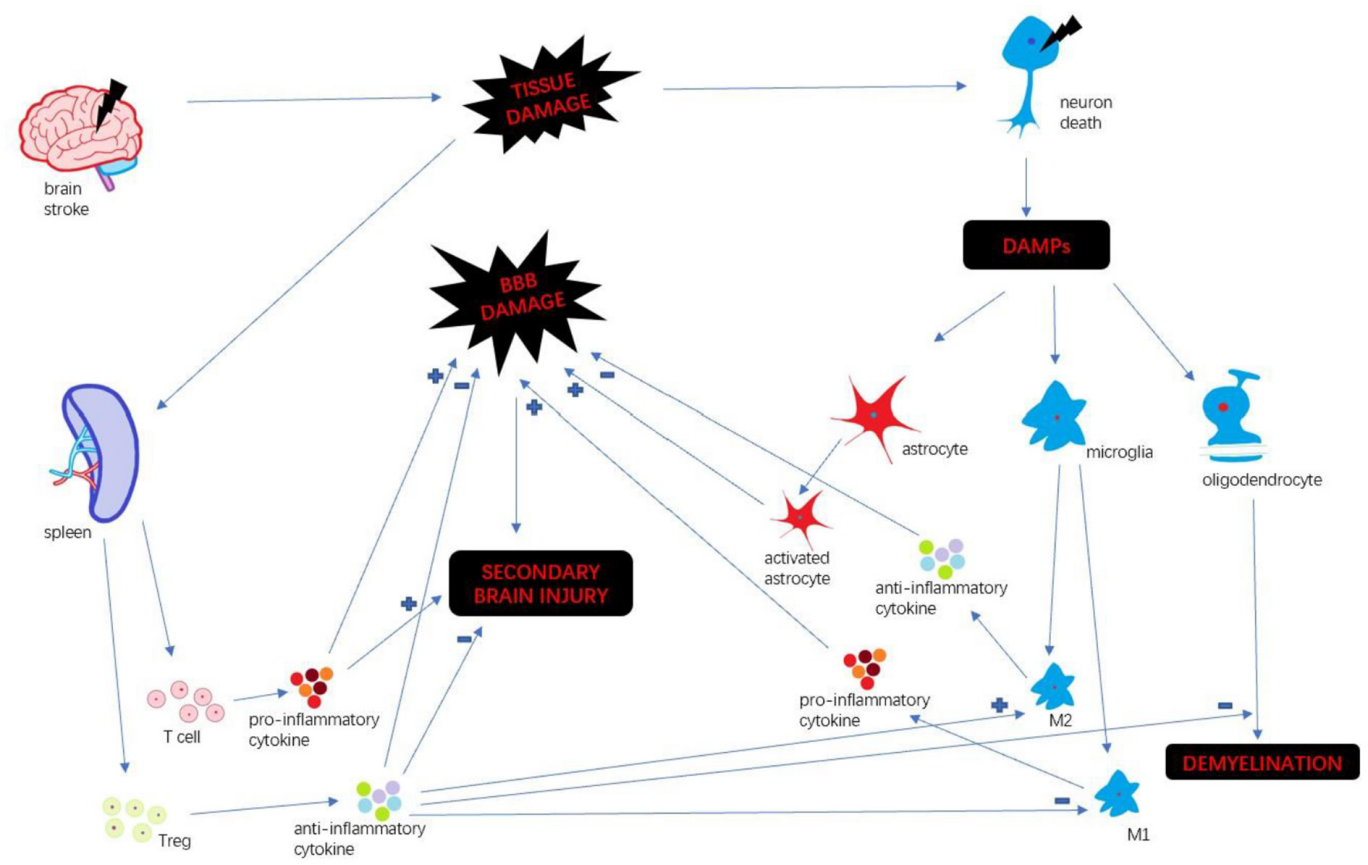
Inflammatory mechanism after stroke.

Stroke is a leading cause of death and long-term disability worldwide (1). Every year ~15 million people suffer from stroke in the world (2). Immune response plays a key factor in stroke progression. Neuroinflammation is an inflammatory response within the central nervous system (CNS), involving many different mediators such as cytokines, chemokines, reactive oxygen species and secondary messengers (3). Oxygen and glucose deprivation following brain tissue damage results in necrosis of neurons and released the different damage-associated molecular patterns (DAMP) which trigger neuroinflammation (4). DAMP include a wide variety of endogenous molecules released on tissue injury, which alter the blood-brain barrier (BBB) permeability, promote peripheral immune cell infiltration, and accelerate tissue edema and brain injury (5). Then microglia are activated and polarize M1 and M2 phenotypes. M1 microglia upregulate a variety of pro-inflammatory mediators which continually damage BBB integrity (6). In the periphery, spleen plays a pivotal role in humoral immunity. Following compromised BBB, spleen releases a mass of peripheral immune cells and inflammatory cytokines infiltrating brain insult. Those different pathways collectively exacerbate the secondary progression of ischemic brain injury (7). We summarize the inflammatory mechanism after stroke (Figure 1).

By now tissue plasminogen activator (tPA) is a proven treatment for acute ischemic stroke (8). However, the use of tPA is restricted by the narrow time window of 4.5 h after ischemic stroke onset, which has limited its use to only a small minority of patients (9). Thrombectomy also is an available approval therapy, which has restricted therapeutic outcome (10). Hence finding a novel effective treatment that could ameliorate the secondary progression of ischemic stroke injury will benefit stroke patients who cannot use tPA (11).

Bone marrow mesenchymal stromal cells (BMSCs) offer an innovative strategy. Stem cell is a kind of special cell which could self-renew, proliferate, and differentiate into specialized cells for cell replacement treatment to stroke (12). Many researches showed that transplanted BMSCs home to sites of injury, which may depend on chemotactic signals (13). Zheng et al. observed that intravenously delivered BMSCs are entrapped in lung microvasculature and are cleared to the liver in 1 day (14). Other researches demonstrated that injected BMSCs preferentially migrate to spleen after stroke (11). Cells through intracerebral transplantation could directly migrate into the infract brain tissue, however, it is more invasive (15). BMSCs take effects through different pathways after stroke, including migrating into ischemic infarction (11), proliferating neuroblasts, replacing impaired cells (16), promoting angiogenesis and neurogenesis (17) and secret a great bunch of neurotrophins. However, BMSCs also cause thrombus and increased intracranial hypertension (15). From many recent researches, except the effects mentioned above, BMSCs could mediate neuroinflammation to accelerate neurofunctional recovery. Therefore, the present review teases out the immunomodulatory effects of BMSCs transplantation after stroke.

## BMSC and central nervous system

With the release of DAMPs following stroke, the microglia become activated, polarizing M1 and M2 phenotypes (18). M1 microglia secrete pro-inflammatory mediators, such as IL-1, IL-6, IL-12, TNF-α, and aggravate brain damage. In contrast, M2 microglia secrete anti-inflammatory cytokines, such as TGF-β and IL-10, accelerating neural repair (19). Stromal derived factor-1 (SDF-1) is mainly produced in microglia/macrophage in a rat middle cerebral artery occlusion (MCAO) model. Shiota et al. found a mesenchymal stem cell (MSC) line (B10) transplantation increased SDF-1 mRNA level from an early time point that persisted until 14 days after MCAO (20). Some researchers found that transplanted BMSCs reduced microglia activation, conferring immunomodulatory effect (21, 22). A study by Nijboer et al. indicated that the number of M2-like (CD206+) microglia was highly increased through intranasal MSC administration (23). In another article, Yang et al. confirmed those findings that BMSCs transplantation promoted M2 phenotype polarization, and decreased the expression of M1 maker *in vivo* and *in vitro* (24). Those researches suggest that BMSCs transplantation could impact M2 polarization meditating inflammatory response.

Astrocytes maintain structure for neurons and contribute to keeping homeostasis of the extracellular environment (25). Also, activated astrocytes play a key participant in neuroinflammation by secreting a large number of inflammatory mediators. The activation of astrocytes could result in dense glial scars, exacerbating neurological deterioration and affecting long-term neuronal recovery (26). Shiota et al. also found B10 transplantation increased the differentiation of neuronal progenitor cells to astrocytes (20). A group of researchers found that BMSCs co-culture enhanced the resistance of astrocytes to hemin neurotoxicity. And they found that BMSCs transplantation promotes astrocytes vimentin expression, and enhance astrocytes antioxidation (26). Zhang et al. co-cultured BMSCs with neurons and astrocytes which exposed to oxygen-glucose deprivation, and found that BMSCs exerted neuroprotection through hindering the apoptosis of neurons and astrocytes (27). Those evidences showed that BMSCs diminished the apoptosis of astrocytes and enhanced its neuroprotection.

Oligodendrocyte precursor cells (OPCs) are immature forms of oligodendrocytes which are essential for repair of damaged white matter after ischemic injury (28). After brain ischemia, immature oligodendrocytes proliferate in the peri-infract areas. Then newly created oligodendrocytes establish contact with un-myelinated axons and form functional myelin sheaths around them (29). BMSCs could reduce the expression of IL-1β protein that could impede the recruitment of OPCs (30). It's reported that BMSCs treatment increased oligodendrogenesis after MCAO, and elevated the number of Nissl-stained neurons in the cortex. Hence, researchers indicated that BMSCs transplantation protects the myelin sheath and promotes axonal restoration (31). In the study by Zarriello et al., OPCs co-cultured with BMSCs increased myelination compared to control group (32). There are some reports that M2 phenotype microglia promoted OPCs differentiation (33). It suggested that BMSCs facilitated OPCs differentiation through promoting M2 phenotype polarization and improved myelination.

## BMSC and peripheral immune system

Spleen is a critical organ in peripheral immune system. After brain damage, spleen could release immune cells and pro-inflammatory mediators which permeate BBB and exacerbate the secondary injuries of cerebral tissue (34). Chiu et al. found that spleen volume decreased over 48 h, then progressively increased following stroke (35). In the research studied by Yang et al., MCAO model rats received human multipotent adult progenitor cells derived from bone marrow. They found that the grafts restored spleen mass reduction (36). Acosta et al. showed that intravenous BMSCs transplantation preferentially migrated to spleen and mitigated inflammation after chronic stroke (11). Our previous study first demonstrated that intracerebral human BMSCs migrated from brain to spleen *via* lymphatic vessels, led by inflammatory signals (37). Those suggested that BMSCs perhaps exert an important role in peripheral immune response *via* spleen.

Following ischemic brain injury, T lymphocytes are activated, infiltrating into damaged brain tissue, and accumulating in the necrotic core (38). Then T cells release many pro-inflammatory cytokines, such as IL-1, IL-6, etc., which induce secondary injuries in the CNS (39). Some researchers demonstrated that T cells also had a detrimental effect on early stroke evolution (40). Oppositely, regulatory T cells (Treg), a special subset of T cells, exert a protective function in neural repair. Much evidence showed that Treg protected compromised BBB (41), intensified white matter repair (42) and promoted M2 microglia polarization to diminish neuroinflammation after stroke (43). Some investigators found that BMSCs with the population of Tregs conferred maximal neuroprotection. In their study, as the immune mediator, the existence of a minority Tregs population within the therapeutic BMSCs population exerted the immunomodulatory and neuroprotective function provided by BMSCs transplantation (44). In another article, Zarriello et al. reported that the native Treg population presented about 0.4% percent of BMSCs, which influenced macrophage polarization toward the more regenerative M2 phenotype. And they cultured oligodendrocyte progenitor cells (OPCs) with BMSCs containing their native Tregs. The result showed that Tregs conferred increased myelination by increasing myelin production (32). The exact molecular mechanisms of how BMSCs influence on Treg is still needed to be further studied.

## BMSC and immunomodulatory molecules

Both central neural cells and peripheral immune cells secret immune factors which play critical roles in central and peripheral system. Immune factors activate inflammatory cascades following cerebral damage (45). Cells transplantation changes the expression of inflammatory cytokines. Few articles systematically summarized the variations of immune factors after BMSCs therapy. Therefore, we reviewed the relevant literature for a summary (Table 1).

**Table 1:** List of important immune factors and their effects.

**Immune factor**	**Effect**	**Outcome after BMSCs transplantation**
IL-1	Pro-inflammation	Decrease (36, 46, 47)
IL-6	Pro-inflammation	Decrease (22, 26, 47)
IL-10	Anti-inflammation	Increase (26, 36, 48)
TNF-α	Pro-inflammation	Decrease (11, 22, 26, 48)
IFN-γ	Pro-inflammation	Decrease (47)
TGF-β	Anti-inflammation	Increase (49)

Interleukin-1 (IL-1) is a typical pro-inflammatory cytokine, first identified as the endogenous pyrogen. The main IL-1 family are IL-1α and IL-1β, which show high sequence homology despite being products of different genes. When brain injury occurred, the up-regulation of IL-1 level were observed (50). Many evidences showed that high levels of IL-1 exacerbated post-stroke damage, though mechanisms involved still unclear (51). Interleukin-6 (IL-6) is identified as a B-cell differentiation factor. IL-6 was observed to be significantly upregulated in Muridae and human patients after stroke (52). Tumor necrosis factor-α (TNF-α) is another important pro-inflammatory cytokine in neuroinflammation. The levels of TNF-α were improved in the damaged brain tissue after an ischemic insult. After brain damage, TNF-α penetrate impaired BBB (53). A number of articles reported the detrimental effects of TNF-α on both glia and neuronal functioning during ischemic stroke (54). As mentioned above, spleen plays a crucial role in neuroinflammation. Interferon gamma (IFN-γ) is associated with the splenic response, which enhances neural injury following middle cerebral artery occlusion (55). The evidences mentioned above unraveled that pro-inflammatory cytokines could lead further cerebral damage.

Salehi et al. found that BMSCs transplanted in rat middle cerebral artery occlusion, resulting down-regulation of IL-1 (46). Huang et al. demonstrated that treated intracerebral hemorrhage rats with BMSCs showed significantly abated expression of IL-1α, IL-6 and IFN-γ (47). In the study by Acosta et al., human BMSCs therapy to MCAO rats reduce TNF-α density (11). Tobin et al. also reported that microglia co-cultured with BMSCs reduced the secretion of IL-6, TNF-α (22). These reports suggested that BMSCs could alleviate inflammation *via* decreasing pro-inflammatory cytokine, such as IL-1, IL-6, TNF-α and IFN-γ.

In contrast, interleukin-10 (IL-10) is a key anti-inflammatory cytokine following ischemic stroke. *In vitro* and *in vivo* models of ischemic stroke showed the neuroprotection of IL-10. Expression of IL-10 in the cerebrum boost neuronal and glial cell survival and dampen of inflammatory responses though a range of signaling pathways (56). Current evidence demonstrated that IL-10 is increased in the brain after stroke (57). Transforming growth factor-β (TGF-β) is another classic anti-inflammatory mediator in brain injury. After stroke, TGF-β was observed in the ischemic brain lesions (58). Many evidence showed that TGF-β mediated microglial phenotype and facilitate neural repair after stroke (59). The finding by Islam et al. demonstrated that TGF-β in ischemic brain exerted sustained anti-inflammatory effects (60). Accordingly, anti-inflammatory cytokines could alleviate inflammatory reaction in the brain.

Liu et al. elucidated that BMSCs treated to MCAO rats increased the expression of IL-10 (48) and Yang et al. confirmed those results (36). In the article by Nakajima et al., BMSCs overexpressing IL-10 exert neuroprotection in acute ischemic stroke (61). Moisan et al. indicated the overexpression of TGF-β in human BMSCs treat MCAO rats (49). These articles supported that BMSCs therapy could ameliorate neuroinflammation though modulating anti-inflammatory cytokine, like IL-10 and TGF-β.

Except mediating immune factors, BMSCs promote angiogenesis and neurogenesis to reduce inflammation by secreting a multitude of growth factors or neurotrophins such as brain-derived neurotrophic factor (BDNF), hepatocyte growth factor (HGF), insulin-like growth factor-1 (IGF-1) and vascular endothelial growth factor (VEGF) (62, 63). Many evidences showed that growth factors have the potential of immunomodulation (64–66). It was reported that HGF therapy could inhibit the disruption of BBB and exert anti-apoptotic and anti-inflammatory effects after cerebral ischemia (67). BDNF signals involved in regulating the production of inflammatory cytokines and oxidative stress (68). IGF-1 could facilitate anti-inflammatory phenotypes on both microglia and astrocytes (69) and decrease the inflammatory cascade (70). And VEGF binds to its receptor to activate downstream signals involved in endothelial activation and vascular inflammation (71). Some researcher found that BMSCs therapy could increase the expression of VEGF and HGF in MCAO model (72, 73). Similarly, Cho et al. observed that the proportions of VEGF-positive cells were higher in the therapy group (74). An article form Li et al. unraveled that concentrations of BDNF and IGF-1, which were mainly derived from transplanted BMSCs, were markedly higher than control group (75). Kim et al. observed similar results (76). Those researches indicated that BMSCs may regulate neuroinflammation through growth factor pathways. However, the exact mechanism still needs to be further investigated.

## Conclusions

To date, growing proof shows the potential for cell replacement therapies to treat stroke. But still many difficulties must be overcome. The precise molecular mechanism of BMSCs treated to stroke is still elusive, which needs to be further studied. Even so, the current studies reported that BMSCs conduct neuroprotective effects after stroke and many patients benefit from it. Immune system is a crucial part to repair the injury. These wide variety of inflammatory pathways may provide new therapeutic targets, thereby giving stroke patients another chance.

## Author contributions

ZW and XW were responsible for drafting of the initial manuscript. YL and GC contributed to the initial draft. KX was the supervisor. All authors contributed to the critical revision of the manuscript.

## Funding

This work was supported by National Natural Science Foundation of China (81901173 and 82060231); Science and Technology Foundation of Guizhou Provincial Health Commission (gzwjkj2021-205).

## Conflict of interest

The authors declare that the research was conducted in the absence of any commercial or financial relationships that could be construed as a potential conflict of interest.

## Publisher's note

All claims expressed in this article are solely those of the authors and do not necessarily represent those of their affiliated organizations, or those of the publisher, the editors and the reviewers. Any product that may be evaluated in this article, or claim that may be made by its manufacturer, is not guaranteed or endorsed by the publisher.

## Abbreviations

BBB: blood-brain barrier
BDNF: brain-derived neurotrophic factor
BMSCs: bone marrow mesenchymal stromal cells
CNS: central nervous system
DAMP: damage-associated molecular patterns
FGF: fibroblast growth factor
HGF: hepatocyte growth factor
IFN-γ: interferon gamma
IGF-1: insulin-like growth factor-1
IL-1: interleukin-1
IL-6: interleukin-6
IL-10: interleukin-10
MCAO: middle cerebral artery occlusion
MSC: mesenchymal stem cell
OPCs: oligodendrocyte precursor cells
SDF-1: stromal derived factor-1
TGF-β: transforming growth factor-β
TNF-α: tumor necrosis factor-α
tPA: tissue plasminogen activator
Treg: regulatory T cells
VEGF: vascular endothelial growth factor.
